# Glycine-extended gastrin enhances somatostatin release from cultured rabbit fundic D-cells

**DOI:** 10.12688/f1000research.2-56.v1

**Published:** 2013-02-20

**Authors:** Ian LP Beales

**Affiliations:** 1 Department of Gastroenterology, Norfolk and Norwich University Hospitals NHS Foundation Trust, Norwich, NR4 7UZ, UK; 2 Norwich Medical School, University of East Anglia, Norwich, NR4 7TJ, UK; 3 Royal Postgraduate Medical School, Hammersmith Hospital, London, W12, UK

## Abstract

The role of the peptide hormone gastrin in stimulating gastric acid secretion is well established. Mature amidated gastrin is processed from larger peptide precursor forms. Increasingly these processing intermediates, such as glycine-extended gastrin (G-Gly) and progastrin, have been shown to have biological activities of their own, often separate and complementary to gastrin. Although G-Gly is synthesized and secreted by gastric antral G-cells, the physiological functions of this putative mediator are unclear. Gastrin and cholecystokinin (CCK) stimulate the secretion of somatostatin from gastric D-cells as part of the feedback control of gastric acid. In this study the effect of G-Gly and gastrin on the release of somatostatin from rabbit fundic D-cells was examined. D-cells were obtained by collagenase-EDTA digestion and elutriation and cultured for 48 hours. With a 2 hour exposure to the peptides, gastrin but not G-Gly stimulated somatostatin release. Treatment of D-cells for 24 hours with gastrin or G-Gly individually, significantly enhanced subsequent basal as well as CCK- and GLP-1-stimulated somatostatin release. Twenty four hours exposure to gastrin combined with G-Gly synergistically enhanced basal and agonist-stimulated somatostatin release and cellular somatostatin content. Gastrin and G-Gly may be important in the longer term regulation of D-cell function.

## Introduction

Gastrin is initially synthesized as a larger precursor protein and subsequently processed, via a multi-step pathway, to the classical active carboxyl-terminal amidated peptide ^1^. It has become apparent that some of the processing intermediates may have biological activities of their own. Significant biological effects have been reported for both the larger progastrin precursor peptide and the shorter carboxyl-terminal glycine-extended gastrin (G-Gly), suggesting that these are important pathophysiological mediators. The majority of studies have examined the pathophysiological roles of gastrin precursors in gastrointestinal cancers and considerable data have implicated these peptides as stimulants of proliferation and/or inhibitors of apoptosis in a variety of tissues and cell lines, including Barrett’s oeosphagus and oesophageal adenocarcinoma ^2, 3^, stomach ^4, 5^, pancreas ^6, 7^ and normal and malignant colonic epithelium ^8– 12^. Growth promoting effects of G-Gly on lung cancer have also been reported ^13^.

Although the precise cell signaling pathways activated by gastrin-processing intermediates have not been definitively described, it seems in most cases that mechanisms distinct from the classical gastrin (CCK2) receptor are involved ^3, 6, 10^. It is not yet clear whether these gastrin-processing intermediates have distinct physiological, as opposed to pathophysiological roles. Glycine-extended gastrin is produced and stored in significant amounts in the gastric antrum, has gastrointestinal trophic effects and interacts with amidated gastrin to modulate gene expression and gastric acid secretion ^14– 16^. Gastrin stimulates both acid secretion and somatostatin release as a feedback inhibitory mechanism ^17^. Similarly, release of cholecystokinin (CCK) from duodenal I-cells is believed to be an important negative feedback mechanism, leading to the inhibition of acid secretion via the release of somatostatin from D-cells in the gastric body and fundus ^17^. Somatostatin release is also stimulated by several other peptide hormones released from the proximal and distal small bowel (including glucagon-like peptide-1 (GLP-1), secretin and oxyntomodulin ^18^ and these form part of the physiological feedback mechanisms that decrease gastric acid in the post-prandial period. The current study was designed to assess the effects of G-Gly on somatostatin release from D-cells and compare these effects with those of amidated gastrin.

## Materials and methods

New Zealand White rabbits (2–2.5 kg) (Charles River Ltd, Margate, UK) were housed singly in 120 x 60 x 60 cm cages and fed *ad libitum* on rabbit chow (Special Diets Services, Witham, UK) with a standard 16/8 hour light/dark cycle according to standard Royal Postgraduate Medical School policy. Rabbits were humanely euthanized with 100 mg/kg pentobarbitone intravenously according to institutional policy. One rabbit was used per cell preparation procedure. Post-mortem, the stomach was removed and primary rabbit fundic D-cells were isolated by EDTA-collagenase digestion and enriched by centrifugal elutriation as described previously ^18– 20^. The D-cell enriched fraction was suspended in culture medium (DMEM: Ham’s F12 50:50 containing 4% foetal calf serum (Gibco, Paisley, UK), 10 mM HEPES pH 7.4, 2 mM glutamine, 8 mg/l bovine insulin, 1 mg/l hydrocortisone, 100 mg/l penicillin, 100 mg/l streptomycin, 100 mg/l gentamicin (all from Sigma, Poole, UK) and plated at 1 x 10 ^6^ cells/well onto 12 well tissue culture plates coated with growth factor-reduced Matrigel (diluted 1:7 with water) (Universal Biologicals, London, UK). Cells were cultured for 48 hours after which either somatostatin release experiments were performed or the culture medium was changed and supplemented with 10 nM gastrin or 10 nM G-Gly as appropriate for a further 24 hours, until release experiments were performed.

Somatostatin release experiments were performed as previously described ^18– 20^: the culture medium was removed, the cells washed, with release medium (Earl’s balanced salt solution containing 0.1% bovine serum albumin and 10 mM HEPES, pH 7.4) and basal somatostatin, as well as 10 nM cholecystokinin (CCK) , and 10 nM glucagon-like peptide-1 (7-36 amide) (GLP-1)-stimulated somatostatin release was assessed over 2 hours ^18– 20^. Cellular somatostatin was extracted by boiling the adherent cells in 3% (final vol/vol) glacial acetic acid in distilled water ^20^. Both released and cellular somatostatin were assessed by radioimmunoassay using K2 anti-somatostatin serum (kindly provided by Professor SR Bloom and Dr M Ghatei, Royal Postgraduate Medical School, Hammersmith Hospital, using ^125^I somatostatin-14 as tracer and human somatostatin-14 as standard (Bachem, St Helens, UK)) as previously described ^18, 20^. Each experimental condition was tested in duplicate and compared with control, untreated wells on the same plate. Results were compared by analysis of variance and Student’s t-test and represent mean ± SEM of 8 different cell preparations. Gastrin (1–17)-Gly (G-Gly) was purchased from NeoMPS (Strasbourg, France), human gastrin-17, sulfated CCK-8 and GLP-1 (7–36) amide were from Bachem.

Cell viability following prolonged gastrin and G-Gly treatment was assessed using the modified 3-(4,5-dimethylthiazol-2-yl)-2,5-diphenyl tetrazolinium bromide (MTT) (Sigma) as previously described ^20^.

## Results

**Figure 1.**
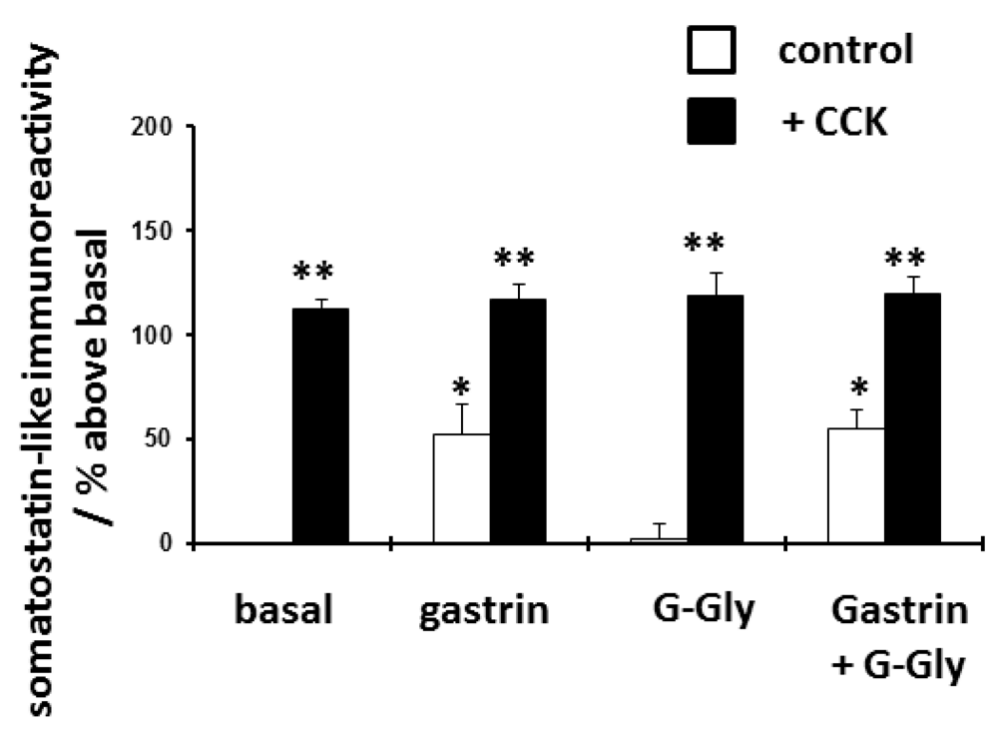
Effect of gastrin (10 nM), glycine-extended gastrin (G-Gly) (10 nM) or both peptides on basal and CCK(10 nM)-stimulated somatostatin release from D-cells. D-cells were cultured for 48 hours and then stimulated with peptides for 2 hours as shown, Somatostatin-like immunoreactivity released into the media was quantified by radioimmunoassay. Results expressed and mean ± SEM, compared to untreated control cells, n = 8, * p<0.05 compared to basal control, *P<0.01 compared to basal control.

Initial experiments with only the standard 2-hour stimulation period (without any prolonged pretreatment with any peptides) confirmed that gastrin increased basal but not CCK-stimulated somatostatin release. G-Gly over the 2 hour stimulation period did not alter basal, gastrin or CCK-stimulated release ( Figure 1 and Table 1). Gastrin alone did stimulate somatostatin release but was less effective than CCK and neither gastrin nor the gastrin plus G-Gly combination had any effect on CCK-stimulated gastrin release.

**Table 1.** Experimental data showing somatostatin-like immunoreactivity (SLI) released from cultured rabbit fundic D-cells stimulated for 2 hours with gastrin (10 nM), glycine-extended gastrin (G-Gly) (10 nM) or both peptides. Experimental data from 8 separate stomach preparations showing somatostatin-like immunoreactivity released from cultured rabbit fundic D-cells stimulated for 2 hours with gastrin, glycine-extended gastrin or both peptides (all 10 nM) +/- CCK (10 nM). SLI results expressed as% of basal, unstimulated release in the relevant stomach preparation.

	Basal		Gastrin 10 nM		G-Gly 10 nM		Gastrin & G-Gly	
Preparation no.	Control	CCK-stimulated	Control	CCK-stimulated	Control	CCK-stimulated	Control	CCK-stimulated
**1**	100	225	154	250	98	253	135	235
**2**	100	235	133	207	103	197	162	241
**3**	100	205	205	220	107	229	207	195
**4**	100	173	154	256	98	167	162	200
**5**	100	243	142	198	106	255	137	257
**6**	100	205	122	206	98	211	130	203
**7**	100	220	182	199	98	216	174	218
**8**	100	215	125	198	105	225	140	210

**Figure 2.**
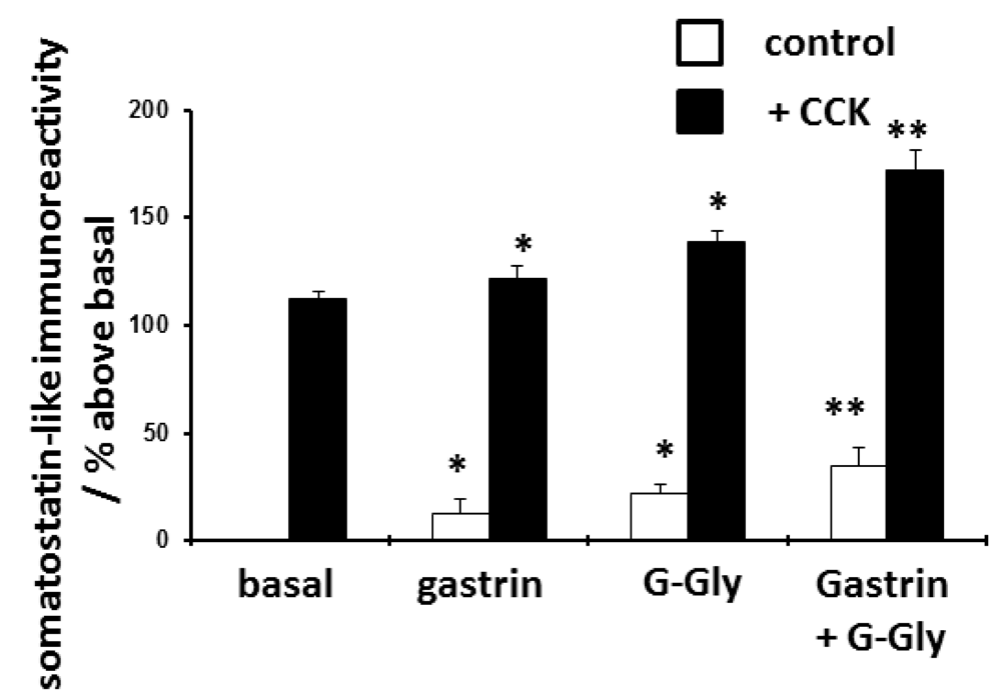
Effects of 24 hour pretreatment with gastrin (10 nM), glycine-extended gastrin (G-Gly) (10 nM) or both peptides on subsequent basal and CCK(10 nM)-stimulated somatostatin release from D-cells. D-cells were cultured for 48 hours and then treated for a further 24 hours with peptides as shown, before stimulation with either CCK or control culture medium for 2 hours. Somatostatin-like immunoreactivity released into the media was quantified by radioimmunoassay. Results expressed and mean ± SEM, compared to untreated control cells, n = 8, * p<0.05 compared to relevant basal control, **p<0.05 compared to gastrin or G-Gly as sole pre-treatment.

Twenty four hours pretreatment with gastrin enhanced subsequent basal somatostatin release by 13% and CCK-stimulated release by 10% (both P<0.05). G-Gly enhanced basal somatostatin release by 22% and CCK-stimulated release by 24% (both p<0.05) ( Figure 2). The combination of gastrin and G-Gly synergistically increased both basal somatostatin release (35%) and subsequent CCK-stimulated somatostatin release (53%) (p<0.05 compared to the effect of either peptide alone) ( Figure 2 and Table 2).

**Table 2.** Experimental data showing somatostatin-like immunoreactivity (SLI) released from cultured rabbit fundic D-cells treated for 24 hours with gastrin (10 nM), glycine-extended gastrin (G-Gly) (10 nM) or both peptides. Experimental data from 8 separate stomach preparations showing somatostatin-like immunoreactivity released from cultured rabbit fundic D-cells stimulated for 2 hours with CCK or GLP-1 (both 10 nM), following a 24-hour pretreatment period with gastrin, glycine-extended gastrin or both peptides (all 10 nM). SLI results expressed as% of basal, unstimulated and untreated release in the relevant stomach preparation.

24h pretreatment	Basal			Gastrin			G-Gly			Gastrin & G-Gly		
2 hr stimulation	Control	CCK	GLP-1	Control	CCK	GLP-1	Control	CCK	GLP-1	Control	CCK	GLP-1
Preparation no.												
**1**	100	226	165	107	229	177	117	251	192	134	290	205
**2**	100	230	187	114	233	188	105	248	189	136	263	195
**3**	100	201	130	118	195	151	120	219	158	152	282	187
**4**	100	182	130	108	185	144	125	203	153	122	239	189
**5**	100	225	144	116	221	143	109	251	159	129	299	184
**6**	100	201	169	120	201	175	129	268	178	125	295	185
**7**	100	221	158	115	225	178	135	254	185	133	269	199
**8**	100	210	165	104	205	198	133	225	201	144	242	223

**Figure 3.**
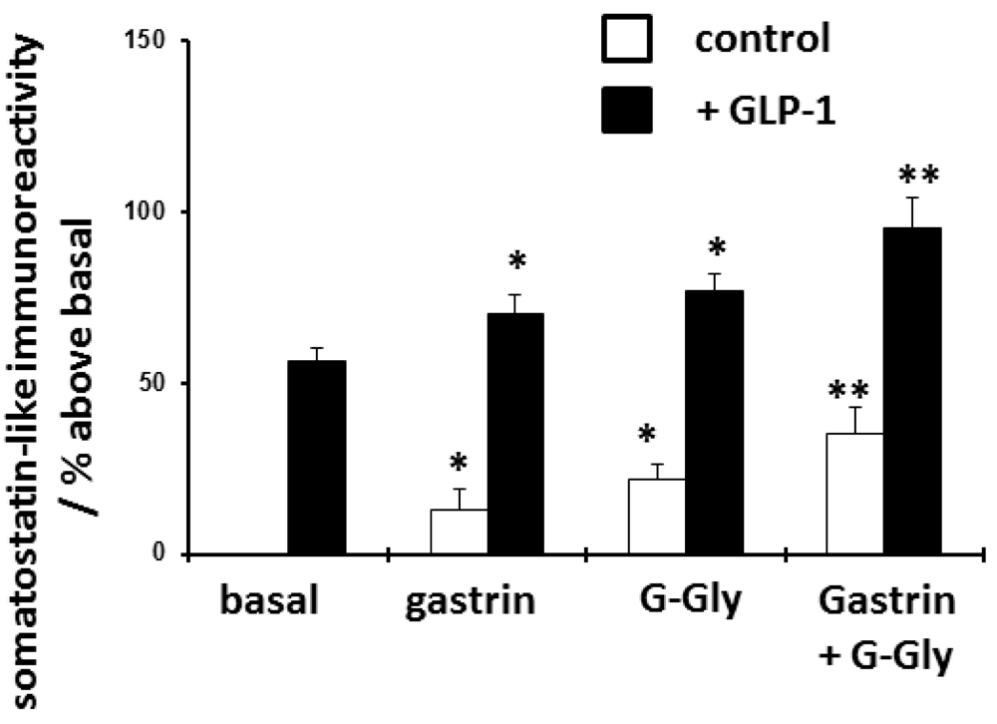
Effect of 24 hour pretreatment with gastrin (10 nM), glycine-extended gastrin (G-Gly) (10 nM) or both peptides on subsequent basal and GLP-1 (10 nM)-stimulated somatostatin release from D-cells. D-cells were cultured for 48 hours and then treated for a further 24 hours with peptides as shown, before stimulation with either GLP-1 or control culture medium for 2 hours. Somatostatin-like immunoreactivity was extracted from cells and quantified by radioimmunoassay. Results expressed and mean ± SEM, compared to untreated control cells, n = 8, * p<0.05 compared to relevant basal control, **p<0.05 compared to gastrin or G-Gly as sole pretreatment.

To further examine the effects of G-Gly pretreatment on agonist-stimulated release, an alternative direct stimulant of rabbit fundic D-cells, GLP-1, was used ^18^. In keeping with previous studies, GLP-1 did stimulate somatostatin release but was markedly less potent than CCK. As shown in ( Figure 3 and Table 2), again 24 hours pretreatment with gastrin alone significantly but relatively modestly potentiated subsequent GLP-1-stimulated somatostatin release (a 25% increase compared to the control GLP-1 treated cells), whilst G-Gly was more effective in enhancing GLP-1-stimulated somatostatin release (a 37% increase compared to the control GLP-1 treated cells). The combination of G-Gly and gastrin was significantly more potent than either peptide alone in enhancing GLP-1 stimulated somatostatin release (a 70% increase compared to the control GLP-1 stimulated cells).

**Figure 4.**
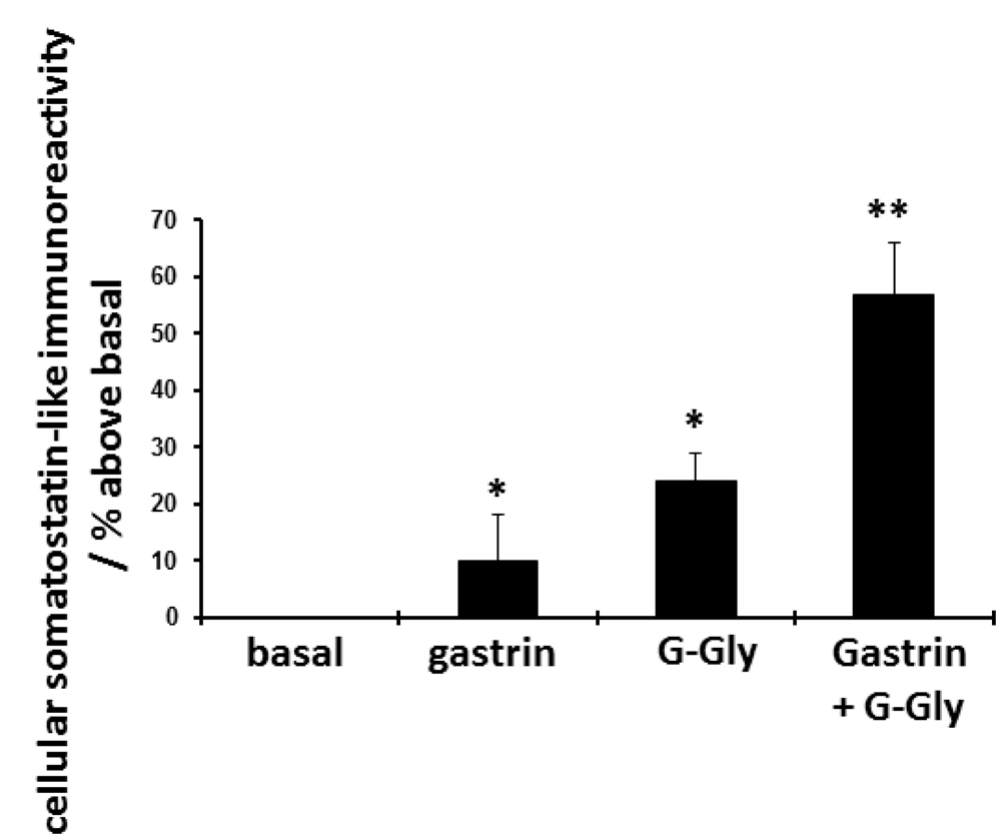
Effect of 24 hour pretreatment with gastrin (10 nM), glycine-extended gastrin (G-Gly) (10 nM) or both peptides on cellular somatostatin content. D-cells were cultured for 48 hours and then treated for a further 24 hours with peptides as shown, Somatostatin-like immunoreactivity was extracted from cells and was quantified by radioimmunoassay. Results expressed and mean ± SEM, compared to untreated control cells, n = 8, *p<0.05 compared to untreated basal control. **p<0.05 compared to gastrin or G-Gly as sole treatment.

Twenty four hour pretreatment with both gastrin peptides individually increased D-cell somatostatin content ( Figure 4 and Table 3). The dual peptide combination was again synergistic in enhancing cellular somatostatin content, the combination increasing total somatostatin levels by 57% compared to untreated basal levels.

**Table 3.** Experimental data showing cellular somatostatin-like immunoreactivity (SLI) in cultured rabbit fundic D-cells treated for 24 hours with gastrin (10 nM), glycine-extended gastrin (G-Gly) (10 nM) or both peptides. Experimental data from 8 separate stomach preparations showing cellular somatostatin-like immunoreactivity contained in cultured rabbit fundic D-cells treated for a 24-hour pretreatment period with gastrin, glycine-extended gastrin or both peptides (all 10 nM). SLI results expresses as% of untreated control cells from the relevant stomach preparation.

Preparation no.	Basal	Gastrin	G-Gly	Gastrin & G-Gly
**1**	100	108	127	149
**2**	100	128	135	193
**3**	100	105	118	160
**4**	100	109	122	152
**5**	100	112	148	192
**6**	100	108	110	129
**7**	100	110	120	150
**8**	100	105	116	143

## Discussion

This study demonstrates that both gastrin and G-Gly enhance the subsequent basal and CCK or GLP-1 stimulated release of somatostatin from rabbit fundic D-cells. No acute stimulatory effects of G-Gly were demonstrated but the 24 hour exposure of D-cells to G-Gly significantly increased somatostatin release. It is clear that hormone release is regulated at multiple points (transcription, translation, processing) ^21^ and that different agents may regulate overall function with different temporal patterns. However, it is not yet clear at which point(s) G-Gly regulates somatostatin release. The increase in cellular somatostatin seen after treatment with G-Gly suggests that upregulation of transcription or translation could be involved. An alternative but not mutually exclusive hypothesis is that the gastrin peptides are specific trophic factors for D-cells and the increased somatostatin release in cultured cells reflects these effects. There was no difference in cell viability, assessed by the modified MTT assay between gastrin or G-Gly treated and non-treated cells, but this does not exclude more subtle enhancement of function. Further studies will be required to elucidate the mechanisms underlying these effects of G-Gly and gastrin.

Previous studies have suggested that G-Gly has important roles in cell proliferation and regulation of acid secretion ^14– 16^, although specific effects on regulation of the gastric endocrine system have not been investigated previously. The effect of G-Gly in increasing acid secretion from cultured parietal cells is only seen with more prolonged exposure ^15^, similar to the results reported here, suggesting this peptide may have longer term regulatory actions on transcription or processing instead of just stimulating hormone release.

It is known that multiple feedback loops regulate gastric acid secretion. Gastrin stimulates both acid secretion and negative feedback inhibition of acid secretion via the simultaneous release of somatostatin ^17^. G-Gly is also released from G-cells following meals and may enhance acid secretion ^22^. The results reported here suggest that longer term exposure of D-cells to gastrin and G-Gly also stimulates a further negative feedback loop by enhancing subsequent somatostatin release thus providing a means to restrain acid hypersecretion caused by hypergastrinaemia and control longer term acid secretion. Several studies have confirmed that G-Gly is produced in gastric antral G-cells ^22– 25^ and this study adds further support to the suggestion that these peptide processing intermediates may have a role in the normal physiological control of the gastric secretions.

The mechanisms of action of both gastrin and G-Gly in enhancing somatostatin release in these circumstances remain to be elucidated. Interestingly, the combination of these peptides had a synergistic effect on the release of somatostatin as has been noted in the control of acid secretion and cell growth ^2– 4, 6, 26– 28^. Gastrin and G-Gly have separate but complimentary actions on cell signaling pathways and gene transcription ^2, 3, 14, 27^. This further supports the notion that both gastrin and G-Gly produced by the gastric antrum and duodenum have important roles in the regulation of gastric homeostasis.
