# Supplementary material for: Glycine-extended gastrin enhances somatostatin release from cultured rabbit fundic D-cells
Source: F1000Res. 2013 Feb 20;2:56. [Version 1] doi: 10.12688/f1000research.2-56.v1 (PMC3814918; doi:10.12688/f1000research.2-56.v1)
Supplement: Peer review history

# Peer review: Referee response for version 1

**Published:** 2013-04-16

**Authors:** Mark Pritchard

**Affiliations:** 1 Department of Gastroenterology, Institute of Translational Medicine, University of Liverpool, Liverpool, UK

The author has assessed the effects of gastrin and glycine-extended gastrin upon somatostatin release from rabbit fundic D cells and this investigation follows on from previous reports by the same author in which he has assessed the effects of other stimulants on somatostatin release using this experimental model. Gastrin (but not G-Gly) had an acute effect following 2h treatment, but both peptides appeared in some way to prime the cells after 20h incubation, so that basal and agonist-stimulated somatostatin release were increased. 

The concentrations of gastrin peptides used in these experiments were high (10nM) and it would therefore be interesting to investigate whether lower concentrations of Gastrin and G-Gly also exerted similar effects in this experimental system. It would be worth referencing the paper which confirmed high homology between the amino acid sequences of human and rabbit gastrin-17, as human peptides were used in this study. It would also be interesting to investigate whether other gastrin precursors (eg progastrin) caused similar effects.

I have read this submission. I believe that I have an appropriate level of expertise to confirm that it is of an acceptable scientific standard.

# Peer review: Referee response for version 1

**Published:** 2013-02-25

**Authors:** Denis McCarthy

**Affiliations:** 1 Division of Gastroenterology, The University of New Mexico, Albuquerque, NM, USA

I have read this submission. I believe that I have an appropriate level of expertise to confirm that it is of an acceptable scientific standard.
